# Commentary on “Rapid identification of *Streptococcus* and *Enterococcus* species using diffuse reflectance-absorbance Fourier transform infrared spectroscopy and artificial neural networks”

**DOI:** 10.1093/femsle/fnx018

**Published:** 2017-01-27

**Authors:** Royston Goodacre, Douglas B. Kell

**Affiliations:** 1 School of Chemistry, The University of Manchester, Manchester, Manchester M1 7DN, UK; 2 Manchester Institute of Biotechnology, The University of Manchester, Manchester, Manchester M1 7DN, UK; 3 Centre for the Synthetic Biology of Fine and Speciality Chemicals (SYNBIOCHEM), The University of Manchester, 131 Princess St, Manchester M1 7DN, UK

**Keywords:** FT-IR spectroscopy, chemometrics, neural networks, machine learning

## Abstract

This is an invited review/commentary by the first and last authors of a paper that was the most cited in *FEMS Microbiology Letters* for 1996, presently showing in excess of 150 citations at Web of Science, and over 200 at Google Scholar. It was the first paper in which diffuse reflectance absorbance FT-IR spectroscopy was used with a supervised learning method in the form of artificial neural networks, and showed that this combination could succeed in discriminating a series of closely related, clinically relevant, Gram-positive bacterial strains.

## Teaser

A 20-years-on review of the most-cited article from the journal in the year 1996.

## INTRODUCTION AND BACKGROUND

Goodacre and Kell had long been seeking phenotypic methods for what is commonly known as ‘rapid microbiology’, a term that covers microbiological methods designed to detect and speciate organisms in various samples (and often to establish their sensitivity or otherwise to antibiotics). Such methods would also be of use in taxonomy. As we stated in the opening of the paper (Goodacre *et al.*^1996^), ‘The ideal method for the examination of the relationships between bacterial strains would have minimum sample preparation, would analyse samples directly (i.e. would not require reagents), would be rapid, automated, non-invasive, quantitative and (at least relatively) inexpensive (Goodacre and Kell 1996)’.

Following one of its multiple renaissances (Rumelhart, McClelland and The PDP Research Group 1986) (a much greater one is presently in progress; LeCun, Bengio and Hinton 2015; Schmidhuber 2015), Kell had become impressed by the ability of artificial neural networks (ANNs) to ‘learn’ to analyse complex multivariate data, and to ‘generalise’ so as to be able to predict the properties of novel, unseen samples. This essentially involved an approach to multivariate calibration, whether the output was quantitative or a classification, and was recognised as a ‘supervised’ method in which the ANNs would be trained or calibrated with samples for which the answer was known. A powerful theorem (Hornik, Stinchcombe and White 1989) had proven that a suitable network (of ‘arbitrary’—hence possibly unfeasible—size) could affect any such non-linear mapping. This meant (in principle) that, given suitable data, an ANN could theoretically solve any classification or regression problem.

In *ca* 1991, Kell had been funded by the Biotechnology Directorate of the UK Science and Engineering Research Council, with Horizon Instruments and Neural Computer Sciences, as part of its LINK Scheme in Analytical Biotechnology, to explore the utility of a combination of pyrolysis mass spectrometry (PyMS) and ANNs to provide analyses of complex biological samples. Pyrolysis involves the thermal breakdown of materials in an inert atmosphere, and while it does not sound as though it might be very reproducible, the use of Curie-point heating meant that the pyrolysis was done at a ‘highly’ reproducible temperature and thus the only bonds to break were those labile at temperatures below the Curie point of the metal or alloy. The fragments would then be sent to a low- (unit mass-) resolution mass spectrometer and provide a pattern or fingerprint that could be analysed. Masses collected were from 51–200 mass:charge, so that the instrument effectively produced 150-dimensional data, perfect for the ANNs of the day (given the computational power then available to us). Goodacre had just finished his PhD at Bristol on the PyMS of various bacteria (e.g. Goodacre and Berkeley 1990), and accepted the postdoctoral position that came with this project. The combination proved extremely successful (∼15 publications from the project) (Goodacre and Kell 1996) and Goodacre secured a Wellcome Trust Career Development Fellowship, in the same Department as Kell in Aberystwyth, to pursue PyMS and ANNs for rapid microbiology. Some highlights of the original project included methods for detecting olive oil adulteration (Goodacre, Kell and Bianchi 1992; Goodacre, Kell and Bianchi 1993), recombinant protein expression (Goodacre *et al*. 1994a) and metabolite overproduction during fermentations (Goodacre *et al*. 1994b, 1995). The latter paper actually used linear transfer functions (Goodacre *et al.*^1995^) to enable extrapolation, now seen as a key element of the much more recent success in training ‘deep’ neural networks (Nair and Hinton 2010; Dahl, Sainath and Hinton 2013).

Based on the success of this basic strategy, Kell had recognised that another means of rapid microbiology might involve an FT-IR instrument ‘flying’ over samples held on a roughened metal plate, where the interrogating light passed through the sample, was reflected by the plate and detected above the plate (in practice, the plate moved, as in a TLC plate scanner). This was funded through another LINK scheme, now by the SERC Chemicals and Pharmaceuticals Directorate, in collaboration with Bruker Instruments UK. It became apparent to us that GSK, who were interested in screening microbial strains for high natural product titres, might like other (bio)pharmaceutical companies be interested in whole-organism phenotyping methods. Kell's long-time collaborator Jem Rowland, from the Aberystwyth Computer Science Department, brought additional numerical heft, while Bruker produced a special instrument for us (based on a TLC plate scanner), which they were subsequently able to sell (after some modifications, in particular using transmission rather than reflective optics). Our use of FT-IR in microbiology was not at all new, this having been pioneered in particular by Naumann and colleagues (Naumann, Fijala and Labischinski 1988; Helm *et al.*^1991^), but our innovations were the use of reflectance spectroscopy—which permitted extremely rapid sample changing—and ANNs (with their much greater analytical power). Subsequently (Winson *et al.*^1997^; Kell *et al.*^1998^), we contrived the name DRASTIC (Diffuse Reflectance Absorbance Spectroscopy Taking In Chemometrics) to describe this combination.

## THE PAPER ITSELF

As in most of our papers using multivariate spectroscopies, it became obvious that supervised methods—in which we calibrate or train the computational (‘machine learning’) system with samples for which the answer is known—were massively more powerful than were ‘unsupervised’ or ‘clustering’ methods, which were really only fit for exploratory analyses. The basic reason is that the latter cannot discriminate signal from noise. This paper was no different. As part of his fellowship, Goodacre had secured the services of Éadaoin Timmins as a PhD student, and had teamed up with Dr Paul Rooney, Head of Microbiology at the local hospital in Aberystwyth, and was eager to try the FT-IR method on what was then a slow and difficult taxonomic problem in terms of speciating various Gram-positive pathogens in the hospital, which could then be used for epidemiology. Those chosen were *Enterococcus faecalis*, *Streptococcus pyogenes*, *S. pneumoniae*, *S. mitis*, *S. bovis* and *E. faecium*. The diffuse-reflectance method was relatively little known, though had been shown in previous work (Mitchell 1993) to generate excellent and reproducible spectra, and it did so in the paper. Even with pre-processing, principal components analysis (an unsupervised method) could not fully discriminate the strains, but when the ‘values’ of the principal components were fed into ANNs as the inputs (thereby reducing the dimensionality and speeding up the training hugely), the ANNs were fully able to discriminate these strains in ‘unseen’ samples. That was the essential finding of the paper.

## WHERE ARE THEY NOW?

Goodacre and Kell both moved to the University of Manchester in 2002/2003 where they hold Chairs, and work in the Manchester Institute of Biotechnology (http://mib.ac.uk). Kell focuses on microbial dormancy Kell focuses on microbial dormancy (Kell, Potgieter and Pretorius 2015; Kell and Kenny 2016), synthetic biology (Currin *et al.*^2014^, ^2015^; Swainston *et al.*^2014^) and pharmaceutical drug transporters (Kell and Goodacre 2014; Kell and Oliver 2014; Kell *et al.*^2015^). Goodacre focuses on Raman spectroscopy (Ashton, Hollywood and Goodacre 2015; Muhamadali *et al.*^2015^) and an interesting variant called surface-enhanced Raman scattering (SERS) (Jarvis and Goodacre 2004; Subaihi *et al.*^2016^; Westley *et al.*^2016^), as well as mass spectrometry-based metabolomics (Goodacre *et al.*^2004^; Dunn *et al.*^2011^; Sayqal *et al.*^2016^). Rowland has retired. Timmins is a Senior Technical Officer at NUI Galway where she supports staff and students in many analytical methods including FT-IR spectroscopy. Rooney is a consultant microbiologist in Belfast City Hospital and he and RG have very recently published together assessing Raman, FT-IR and MALDI-TOF-MS for rapid subspeciation of *Enterococcus faecium* (AlMasoud *et al.*^2016^), illustrating that rapid discrimination within this species of bacterium is still very important, even 20 years on.

## WHAT HAPPENED SUBSEQUENTLY?

We have continued to develop phenotypic methods for rapid microbiology (e.g. Davey and Kell 1996; Goodacre *et al.*^1998^), including in this journal (Goodacre, Heald and Kell 1999). Recognising the power of whole-cell phenotyping methods, which were shortly to be popularised as ‘omics‘, Goodacre and Kell went into metabolomics, with the first paper using the word ‘metabolome’ (Oliver *et al.*^1998^) (commentary; Kell and Oliver 2016) showing FT-IR spectra from the same instrument, and Goodacre being Founding Editor of the eponymous journal. Recent examples of our metabolomics work include Begley *et al.* (2009); Zelena *et al.* (2009); Dunn *et al.* (2011, 2015), while Goodacre has also published widely using FT-IR, Raman and SERS (e.g. reviews Ellis and Goodacre 2006; Jarvis and Goodacre 2008; Huang *et al.*^2010^; Ellis *et al.*^2012^) as well as mass spectrometry (e.g. Dunn *et al.*^2010^; Rattray *et al.*^2014^), including volatile analysis for non-invasive human infections (Fowler *et al.*^2015^).

## WHY SO HIGHLY CITED?

In one sense, it is recognised that ‘analytical methods’ can be highly cited (e.g. those for protein content; Lowry *et al.*^1951^; Bradford 1976), and looking through the citing papers, most are either on metabolomics or on rapid microbiology. Arguably the main reason for the paper being highly cited was at least partly its pioneering primacy, but mainly that the paper did deliver precisely what it set out to do, i.e. it did indeed provide a method for rapid microbiology that ‘would have minimum sample preparation, would analyse samples directly (i.e. would not require reagents), would be rapid, automated, non-invasive, quantitative and (at least relatively) inexpensive’ (Goodacre *et al.*^1996^).

## References

[bib1] AlMasoud N, Xu Y, Ellis DI Rapid discrimination of *Enterococcus**faecium* strains using phenotypic analytical techniques. Anal Meth 2016;8:7603–13.

[bib2] Ashton L, Hollywood KA, Goodacre R Making colourful sense of Raman images of single cells. Analyst 2015;140:1852–8. 25666258 10.1039/c4an02298j

[bib3] Begley P, Francis-McIntyre S, Dunn WB Development and performance of a gas chromatography-time-of-flight mass spectrometry analysis for large-scale non-targeted metabolomic studies of human serum. Anal Chem 2009;81:7038–46. 19606840 10.1021/ac9011599

[bib4] Bradford MM A rapid and sensitive method for the quantitation of microgram quantities of protein utilizing the principle of protein-dye binding. Anal Biochem 1976;72:248–54. 942051 10.1016/0003-2697(76)90527-3

[bib5] Currin A, Swainston N, Day PJ SpeedyGenes: a novel approach for the efficient production of error-corrected, synthetic gene libraries. Protein Eng Des Sel 2014;27:273–80. 25108914 10.1093/protein/gzu029 PMC4140418

[bib6] Currin A, Swainston N, Day PJ Synthetic biology for the directed evolution of protein biocatalysts: navigating sequence space intelligently. Chem Soc Rev 2015;44:1172–239. 25503938 10.1039/c4cs00351a PMC4349129

[bib7] Dahl GE, Sainath TN, Hinton GE Improving deep neural networks for LVCSR using rectified linear units and dropout. Proc ICASSP 2013:8609–13.

[bib8] Davey HM, Kell DB Flow cytometry and cell sorting of heterogeneous microbial populations: the importance of single-cell analysis. Microbiol Rev 1996;60:641–96. 8987359 10.1128/mr.60.4.641-696.1996 PMC239459

[bib9] Dunn WB, Broadhurst DI, Atherton HJ Systems level studies of mammalian metabolomes: the roles of mass spectrometry and nuclear magnetic resonance spectroscopy. Chem Soc Rev 2010;40:387–426. 20717559 10.1039/b906712b

[bib10] Dunn WB, Broadhurst D, Begley P Procedures for large-scale metabolic profiling of serum and plasma using gas chromatography and liquid chromatography coupled to mass spectrometry. Nat Protoc 2011;6:1060–83. 21720319 10.1038/nprot.2011.335

[bib11] Dunn WB, Lin W, Broadhurst D Molecular phenotyping of a UK population: defining the human serum metabolome. Metabolomics 2015;11:9–26. 25598764 10.1007/s11306-014-0707-1 PMC4289517

[bib12] Ellis DI, Goodacre R Metabolic fingerprinting in disease diagnosis: biomedical applications of infrared and Raman spectroscopy. Analyst 2006;131:875–85. 17028718 10.1039/b602376m

[bib13] Ellis DI, Brewster VL, Dunn WB Fingerprinting food: current technologies for the detection of food adulteration and contamination. Chem Soc Rev 2012;41:5706–27. 22729179 10.1039/c2cs35138b

[bib14] Fowler SJ, Basanta-Sanchez M, Xu Y Surveillance for lower airway pathogens in mechanically ventilated patients by metabolomic analysis of exhaled breath: a case-control study. Thorax 2015;70:320–5. 25661115 10.1136/thoraxjnl-2014-206273

[bib15] Goodacre R, Berkeley RCW Detection of small genotypic changes in *Escherichia coli* by pyrolysis mass spectrometry. FEMS Microbiol Lett 1990;71:133–8. 10.1016/0378-1097(90)90045-r 2276603

[bib16] Goodacre R, Kell DB Pyrolysis mass spectrometry and its applications in biotechnology. Curr Opin Biotechnol 1996;7:20–8. 8791308 10.1016/s0958-1669(96)80090-5

[bib17] Goodacre R, Heald JK, Kell DB Characterisation of intact microorganisms using electrospray ionization mass spectrometry. FEMS Microbiol Lett 1999;176:17–24.

[bib18] Goodacre R, Karim A, Kaderbhai MA Rapid and quantitative analysis of recombinant protein expression using pyrolysis mass spectrometry and artificial neural networks - application to mammalian cytochrome b_5_ in *Escherichia coli*. J Biotechnol1994a;34:185–93. 7764850 10.1016/0168-1656(94)90088-4

[bib19] Goodacre R, Kell DB, Bianchi G Neural networks and olive oil. Nature 1992;359:594.

[bib20] Goodacre R, Kell DB, Bianchi G Rapid assessment of the adulteration of virgin olive oils by other seed oils using pyrolysis mass spectrometry and artificial neural networks. J Sci Food Agr 1993;63:297–307.

[bib21] Goodacre R, Timmins ÉM, Burton R Rapid identification of urinary tract infection bacteria using hyperspectral whole-organism fingerprinting and artificial neural networks. Microbiology UK 1998;144:1157–70. 10.1099/00221287-144-5-1157 9611790

[bib22] Goodacre R, Timmins ÉM, Rooney PJ Rapid identification of *Streptococcus* and *Enterococcus* species using diffuse reflectance-absorbance Fourier transform infrared spectroscopy and artificial neural networks. FEMS Microbiol Lett 1996;140:233–9. 8764485 10.1016/0378-1097(96)00186-3

[bib23] Goodacre R, Trew S, Wrigley-Jones C Rapid screening for metabolite overproduction in fermentor broths, using pyrolysis mass spectrometry with multivariate calibration and artificial neural networks. Biotechnol Bioeng1994b;44:1205–16. 18618547 10.1002/bit.260441008

[bib24] Goodacre R, Trew S, Wrigley-Jones C Rapid and quantitative analysis of metabolites in fermentor broths using pyrolysis mass spectrometry with supervised learning: application to the screening of *Penicillium**chryosgenum* fermentations for the overproduction of penicillins. Anal Chim Acta 1995;313:25–43.

[bib25] Goodacre R, Vaidyanathan S, Dunn WB Metabolomics by numbers: acquiring and understanding global metabolite data. Trends Biotechnol 2004;22:245–52. 15109811 10.1016/j.tibtech.2004.03.007

[bib26] Helm D, Labischinski H, Schallehn G Classification and identification of bacteria by Fourier transform infrared spectroscopy. J Gen Microbiol 1991;137:69–79. 1710644 10.1099/00221287-137-1-69

[bib27] Hornik K, Stinchcombe M, White H Multilayer feedforward networks are universal approximators. Neural Networks 1989;2:359–66.

[bib28] Huang WE, Li M, Jarvis RM Shining light on the microbial world the application of Raman microspectroscopy. Adv Appl Microbiol 2010;70:153–86. 20359457 10.1016/S0065-2164(10)70005-8

[bib29] Jarvis RM, Goodacre R Discrimination of bacteria using surface-enhanced Raman spectroscopy. Anal Chem 2004;76:40–7. 14697030 10.1021/ac034689c

[bib30] Jarvis RM, Goodacre R Characterisation and identification of bacteria using SERS. Chem Soc Rev 2008;37:931–6. 18443678 10.1039/b705973f

[bib31] Kell DB, Goodacre R Metabolomics and systems pharmacology: why and how to model the human metabolic network for drug discovery. Drug Discov Today 2014;19:171–82. 23892182 10.1016/j.drudis.2013.07.014 PMC3989035

[bib32] Kell DB, Kenny LC A dormant microbial component in the development of pre-eclampsia. Front Med Obs Gynecol 2016;3:60. 10.3389/fmed.2016.00060 PMC5126693 27965958

[bib33] Kell DB, Oliver SG How drugs get into cells: tested and testable predictions to help discriminate between transporter-mediated uptake and lipoidal bilayer diffusion. Front Pharmacol 2014;5:231. 25400580 10.3389/fphar.2014.00231 PMC4215795

[bib34] Kell DB, Oliver SG The metabolome 18 years on: a concept comes of age. Metabolomics 2016;12:148. 27695392 10.1007/s11306-016-1108-4 PMC5009154

[bib35] Kell DB, Potgieter M, Pretorius E Individuality, phenotypic differentiation, dormancy and ‘persistence’ in culturable bacterial systems: commonalities shared by environmental, laboratory, and clinical microbiology. F1000Research 2015;4:179. 26629334 10.12688/f1000research.6709.1 PMC4642849

[bib36] Kell DB, Swainston N, Pir P Membrane transporter engineering in industrial biotechnology and whole-cell biocatalysis. Trends Biotechnol 2015;33:237–46. 25746161 10.1016/j.tibtech.2015.02.001

[bib37] Kell DB, Winson MK, Goodacre R DRASTIC (Diffuse Reflectance Absorbance Spectroscopy Taking In Chemometrics). A novel, rapid, hyperspectral, FT-IR-based approach to screening for biocatalytic activity and metabolite overproduction. Stud Org Chem 1998;53:61–75.

[bib38] LeCun Y, Bengio Y, Hinton G Deep learning. Nature 2015;521:436–44. 26017442 10.1038/nature14539

[bib39] Lowry OH, Rosebrough NJ, Farr AL Protein measurement with the Folin phenol reagent. J Biol Chem 1951;193:265–75. 14907713

[bib40] Mitchell MB Fundamentals and applications of diffuse reflectance infrared Fourier transform (DRIFT) spectroscopy. ACS Adv Chem Ser 1993;236:351–75.

[bib41] Muhamadali H, Chisanga M, Subaihi A Combining Raman and FT-IR spectroscopy with quantitative isotopic labeling for differentiation of *E. coli* cells at community and single cell levels. Anal Chem 2015;87:4578–86. 25831066 10.1021/acs.analchem.5b00892

[bib42] Nair V, Hinton GE Rectified linear units improve restricted Boltzmann machines. In Proceedings International Conference on Machine Learning. Madison, WI: Omnipress, 2010, 807–14.

[bib43] Naumann D, Fijala V, Labischinski H The differentiation and identification of pathogenic bacteria using FT-IR and multivariate statistical analysis. Mikrochim Acta 1988;1:373–7.

[bib44] Oliver SG, Winson MK, Kell DB Systematic functional analysis of the yeast genome. Trends Biotechnol 1998;16:373–8. 9744112 10.1016/s0167-7799(98)01214-1

[bib45] Rattray NJW, Hamrang Z, Trivedi DK Taking your breath away: metabolomics breathes life in to personalized medicine. Trends Biotechnol 2014;32:538–48. 25179940 10.1016/j.tibtech.2014.08.003

[bib46] Rumelhart DE, McClelland JL, The PDP Research Group (eds). Parallel Distributed Processing: Experiments in the Microstructure of Cognition, Vols I, II Cambridge, MA: MIT Press, 1986.

[bib47] Sayqal A, Xu Y, Trivedi DK Metabolic analysis of the response of *Pseudomonas**putida* DOT-T1E strains to toluene using Fourier transform infrared spectroscopy and gas chromatography mass spectrometry. Metabolomics 2016;12:112. 27398079 10.1007/s11306-016-1054-1 PMC4916193

[bib48] Schmidhuber J Deep learning in neural networks: an overview. Neural Networks 2015;61:85–117. 25462637 10.1016/j.neunet.2014.09.003

[bib49] Subaihi A, Almanqur L, Muhamadali H Rapid, accurate, and quantitative detection of propranolol in multiple human biofluids via surface-enhanced Raman scattering. Anal Chem 2016;88:10884–92. 27731981 10.1021/acs.analchem.6b02041

[bib50] Swainston N, Currin A, Day PJ GeneGenie: optimised oligomer design for directed evolution. Nucleic Acids Res 2014;12:W395–400. 10.1093/nar/gku336 PMC4086129 24782527

[bib51] Westley C, Xu Y, Carnell AJ Label-free surface enhanced Raman scattering approach for high-throughput screening of biocatalysts. Anal Chem 2016;88:5898–903. 27132981 10.1021/acs.analchem.6b00813

[bib52] Winson MK, Goodacre R, Timmins ÉM Diffuse reflectance absorbance spectroscopy taking in chemometrics (DRASTIC). A hyperspectral FT-IR-based approach to rapid screening for metabolite overproduction. Anal Chim Acta 1997;348:273–82.

[bib53] Zelena E, Dunn WB, Broadhurst D Development of a robust and repeatable UPLC-MS method for the long-term metabolomic study of human serum. Anal Chem 2009;81:1357–64. 19170513 10.1021/ac8019366

